# *Candida* Variety in the Oral Cavity of Mexican Subjects with Type 2 Diabetes Mellitus and TLR2 Gene Expression

**DOI:** 10.3390/clinpract14020031

**Published:** 2024-02-27

**Authors:** Nadia Mabel Pérez-Vielma, Modesto Gómez-López, María de los Ángeles Martínez-Godínez, Ana Laura Luna-Torres, Aarón Domínguez López, Ángel Miliar-García

**Affiliations:** 1 Sección de Estudios de Posgrado e Investigación, Centro Interdisciplinario de Ciencias de la Salud Unidad Santo Tomás, Instituto Politécnico Nacional, Mexico City 11340, Mexico; nadiampv@gmail.com (N.M.P.-V.); alunat@ipn.mx (A.L.L.-T.); 2 Laboratorio de Biología Molecular, Sección de Estudios de Posgrado e Investigación, Escuela Superior de Medicina, Instituto Politécnico Nacional, Mexico City 11340, Mexico; mgomezlo@ipn.mx (M.G.-L.); mmartinezgo@ipn.mx (M.d.l.Á.M.-G.); adominguezl@ipn.mx (A.D.L.)

**Keywords:** *Candida*, type 2 diabetes mellitus, PAP stain, oral cavity, molecular diagnosis, TLR2

## Abstract

Background: The aim was to diagnose *Candida* in the oral cavity of subjects with type 2 diabetes mellitus (T2DM) using a genotyping technique and compare the results with those from conventional diagnosis by Papanicolaou (Pap) staining. Methods: Palatal mucosa smears were performed on 18 dental care patients diagnosed with T2DM and grade I, II, and III prosthetic stomatitis who met the inclusion criteria; 18 healthy control subjects were also included in the study. Hemoglobin A1c (HbA1c) levels were determined from total blood. Using exfoliative cytology, the Pap staining technique was used to diagnose candidiasis. Exfoliative cytology was also used for molecular diagnosis; DNA was obtained for *Candida* genotyping, and RNA was used for gene expression studies. Results: Clinical patterns indicated that all subjects were positive for *Candida*; however, Pap analysis revealed only three positive subjects, whereas end-point polymerase chain reaction (PCR) analysis revealed 15 subjects with some type of *Candida*. The most common *Candida* species found were *Candida guilliermondii* (38.8%), *Candida krusei* (33.3%), *Candida tropicalis*, *and Candida lusitaniae* (22.2%). Interestingly, the coexpression of different species of *Candida* was found in various patients. In all patients, HbA1c levels were increased. Gene expression analysis showed a significant decrease (*p* ≤ 0.05) in *TLR2* expression in positive subjects, whereas *TLR4* expression did not differ significantly among patients. Conclusions: The end-point PCR technique showed better sensitivity for the diagnosis of *Candida* when compared with the diagnosis by Pap staining. T2DM subjects showed an increased presence of *C*. *guilliermondii* that was correlated with decreased *TLR2* expression.

## 1. Introduction

*Candida* is a type of fungus present in the oral flora of healthy subjects [1]; it has a wide distribution that includes the gastrointestinal, urinary, and respiratory tracts [2]. Fifteen subspecies of *Candida* cause different diseases in humans; although 90% of subspecies are harmless, the most common pathogenic species are *Candida albicans*, *Candida glabrata*, *Candida tropicalis*, *Candida parapsilosis*, *and Candida krusei* [3]. *Candida* is considered an opportunistic organism that causes oral infections; it is mainly found under conditions of malnutrition, changes in pH, and/or high concentrations of glucose in saliva [4]. The proliferation of the different subspecies of *Candida* is increased in patients with type 2 diabetes mellitus (T2DM) [5,6,7,8] because they present with high levels of glucose, immunological changes, and immunosuppression [9] and changes to their general pH levels induced by their chronic metabolic syndrome [10]. In 2017, according to the WHO and the International Diabetes Federation, the world diabetic population was estimated at 425 million people [11,12]. *Candida* can be found locally or generally [13], but it is characterized by a reduction in saliva when it is found in the mouth [14] as well as microvascular degeneration and neutrophil activity, which are associated with the high levels of glucose found in patients with T2DM [15,16]. In addition, an increase in digestive enzymes has been reported in patients with T2DM and candidiasis [17,18,19], which benefits *Candida* because patients with diabetes suffer a generalized state of immunosuppression [20,21,22]. Two hundred subspecies of *Candida* have been characterized, of which *C. albicans*, *C. glabrata*, *C. parapsilosis*, *C. tropicalis*, and *C. krusei* [23] are most frequently detected in patients with candidiasis. Such species diversity makes *Candida* difficult to treat [24]. Of these subspecies, *C. albicans* is the most aggressive; it is associated with a 93% increase in the development of stomatitis [25]. The immune response of patients with candidiasis is initiated by pathogens associated with molecular patterns, which are recognized by specific receptors of the innate immune response known as pathogen recognition receptors (PRRs) [26,27]. The innate immune response to *C. albicans* occurs through monocytes, neutrophils, macrophages, and dendritic cells; these express PRRs and establish the first line of defense against *C. albicans* infection [26]. *Candida albicans* can be recognized by beta-glucans, dectin-1, and lecithin type C receptors, primarily contained in its membrane, as well as by TLR2 and TLR4 expression in the human immune response [28,29,30]. As part of the *Candida* infection response, a proinflammatory response is triggered by activation of the NLRP3 inflammasome [31,32], which in turn activates IL-1b [33].

Specific diagnoses of oral candidiasis in patients with T2DM are important for improving treatment because resistance to therapeutic procedures is largely due to misdiagnosis. Therefore, the objective of the present study was to identify *Candida* species associated with oral alterations in patients with T2DM using polymerase chain reaction (PCR). In addition, the results of molecular diagnoses were compared with conventional diagnoses obtained using Papanicolaou (Pap) smears. Furthermore, the expression of *TLR2* and *TLR4* genes was evaluated as potential biomarkers of the immune response.

## 2. Materials and Methods

### 2.1. Subjects

This research was approved by the Research Ethics Committee of the IPN School of Medicine (No. ESM.CE-01/01-29-2016). Written informed consent was obtained from all participants. The experiments were performed in accordance with the ethical principles of the Declaration of Helsinki and were consistent with the Good Clinical Practice Guidelines. The participants included 18 adults of both sexes who attended a dental consultation at the Centro Interdisciplinario de Ciencias de la Salud, Unidad Santo Tomas (CICS UST) del Instituto Politécnico Nacional; these patients were ≥40 years old, had been diagnosed with T2DM, and met the inclusion criteria. In addition, 18 healthy adults of both sexes aged ≥40 years were used as a control group. Peripheral venous blood was drawn from each subject in the morning, and the glycated hemoglobin HbA1c was determined. Palatal mucosa smears were performed on patients diagnosed with grade I, II, and III prosthetic stomatitis as well as T2DM. Samples were fixed with absolute ethanol, and staining was performed according to the Pap technique.

### 2.2. Gene Expression Analysis and Genotyping

Total RNA was isolated from smears of the palatal mucosa using TRIzol reagent according to the manufacturer’s instructions (TriPure Isolation Reagent; Roche Applied Science, Indianapolis, IN, USA). The amount and purity of the isolated RNA were quantified nanophotometrically by measuring the optical densities at 260 and 280 nm. The integrity of all samples was confirmed by agarose gel electrophoresis. Before reverse transcription, RNA samples were treated further with amplification-grade DNase I (Invitrogen, Carlsbad, CA, USA) to remove trace amounts of contaminating DNA. All RNA samples were stored in the RNA elution solution at −80 °C. Subsequently, 0.5–1.0 µg of total RNA was subjected to reverse transcription using a First-Strand cDNA Synthesis Kit (Roche Diagnostics, GmbH, Mannheim, Germany) with random hexamer primers in an Eppendorf thermocycler according to the manufacturer’s instructions. Finally, the concentration of the newly generated cDNA was determined nanospectrophotometrically.

Real-time quantitative PCR (qPCR) assays were performed using specific oligonucleotide primers that were generated using online assay design software (https://qpcr.probefinder.com/organism.jsp, accessed on 23 November 2019). The sequences of the primers used to determine gene expression were as follows: *TLR2* forward: 5′-CCT TTG GAT CCT GCT TGC-3′; *TLR2* reverse: 5′-CGT TCT CTC AGG TGA CTG CTC-3′; *TLR4* forward: 5′-TCC ATG CAT TGA TAA GTA ATA TTA GGA-3′; *TLR4* reverse: 5′-CTC TCC TGC GTG AGA CCA G-3′. The sequence of the primers used to determine the genotype of each *Candida* strain with its specific alignment temperature was as follows: *C. albicans*: 5′-AGC TGC CGC CAG AGG TCT AA-3′ (583 bp); *C. glabrata*: 5′-TTG TCT GAG CTC GGA GAG AG-3′ (929 bp); *C. parapsilosis*: 5′-GTC AAC CGA TTA TTT AAT AG-3′ (570 bp); *C. tropicalis*: 5′-GAT TTG CTT AAT TGC CCC AC-3′ (583 bp); *Candida dubliniensis*: 5′-CTC AAA CCC CTA GGG TTT GG-3′ (591 bp); *C. krusei*: 5′-CTG GCC GAG CGA ACT AGA CT-3′ (590 bp); *C. guilliermondii*: 5′-TTG GCC TAG AGA TAG GTT GG-3′ (668 bp); *Candida lusitaniae*: 5′-TTC GGA GCA ACG CCT AAC CG-3′ (433 bp); and a universal reverse primer: 5′-TTC TTT TCC TCC GCT TAT TG-3′. For gene expression analysis, the reaction mixture, containing 1 µL of standard cDNA at an appropriate dilution, was prepared according to the manufacturer’s instructions (Roche Diagnostics, GmbH). A Light Cycler Nano Real-Time PCR System (Roche Diagnostics, Mannheim, Germany) was used for all amplifications, with the following settings used for Universal Probe Library-based assays: an initial denaturation step for 10 min at 95 °C and then 45 cycles of 10 s at 94 °C, 20 s at 60 °C, and 5 s at 72 °C. Each qPCR assay included a standard curve of four serial dilution points for each gene. The mRNA levels were normalized to the expression of the endogenous control, 18S mRNA. All qPCR experiments require standardization of the reaction efficiency curves for gene expression. We calculated the mRNA levels using the comparative parameter threshold cycle (Ct) method. In the standardization formula, 2^−ΔΔCt^, the value 2 corresponds to the qPCR efficiency reaction (dynamic range curve or dilutions of the constitutive gene).

### 2.3. Statistical Analysis

Descriptive statistics were performed, and the expression data are expressed as means ± standard deviations (SD). Data were analyzed using either the unpaired Student’s *t*-test or a one-way analysis of variance. GraphPad Prism version 8.00 for Windows and SPSS were used to conduct the statistical analyses and graph the data.

## 3. Results

### 3.1. Characteristics of the Participants

The clinical characteristics of the 18 patients included in this study are shown in Table 1. In patients with T2DM, the mean age was 63.3 ± 14.2 years. Most patients were female (66.6%), most were nonsmokers, and a high percentage were hypertensive (61.1%). Type I stomatitis was predominant (61.1%) compared with Type II stomatitis (38.8%). When reviewing the clinical patterns, an increase in pseudomembranous candidiasis (83.3%) and a high percentage of glycosylated hemoglobin were observed.

### 3.2. Pap Smear Results

**Figure 1:**
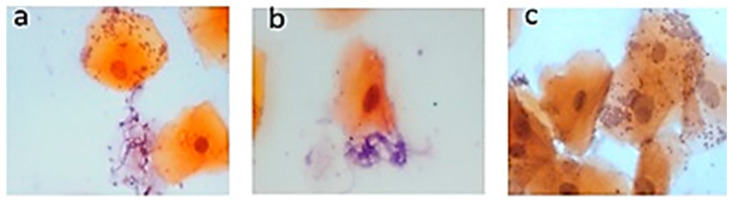
Microscopy of the PAP stain on the palate. Smears were taken from the palatal mucosa of the subjects with stomatitis, and staining with Papanicolaou solution was performed. Elongated hyphae correspond to the fungus Candida (**a**,**b**) and the appearance of round cells that form small groups (**c**) (PAP 60×).

Exfoliative cytology of the samples from patients with T2DM confirmed the presence of *Candida*. Figure 1 shows the hyphae of the *Candida* types. Tangled tubular hyphae were observed related to desquamated epithelial cells (Figure 1a,b), and *Candida* was observed in the form of yeast and has an appearance of round or ovoid cells, which form small groups (Figure 1c).

Identification by end-point PCR results and the use of specific primers for each studied species of *Candida* are shown in Table 2. The most common *Candida* species found were *Candida guilliermondii* (38.8%), *Candida krusei* (33.3%), *Candida tropicalis*, *and Candida lusitaniae* (22.2%). Interestingly, species coexpression was found among the study subjects. The species with the highest coexpression were *C*. *guilliermondii* and *C. krusei.* In four patients, *Candida* species were not found.

### 3.3. Gene Expression of TRL2 and TLR4

**Figure 2:**
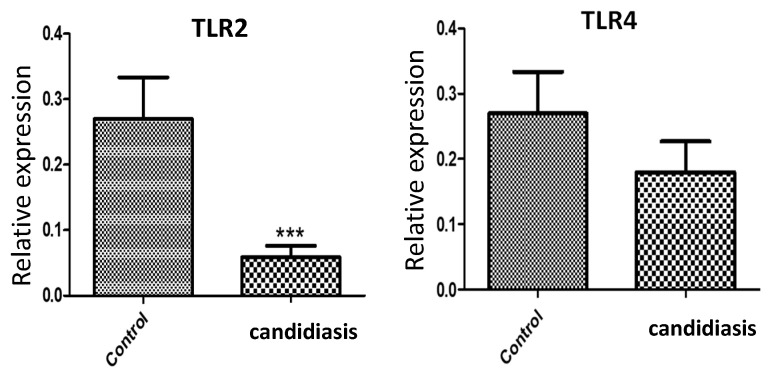
Quantitative real-time PCR assays of the genes encoding TLR2 and TLR4. Samples from smears of the palatal mucosa were collected, and total RNA was extracted as described in Section 2. The relative expression level of each gene was quantified and normalized to 18S ribosomal RNA. Data from patients with candidiasis and control subjects are shown. *** *p* < 0.0001.

In addition, qPCR analysis revealed a significant decrease (*p* < 0.0001) in the mRNA expression of *TLR2* in the candidiasis group relative to that in the control group (Figure 2). In contrast, there was no significant difference in *TLR4* expression between the groups.

## 4. Discussion

Here, the most common variants of *Candida* were identified in the oral cavity of patients with diabetes and candidiasis. When using Pap smears, a conventional method for the diagnosis of *Candida*, it was possible to detect 3 of 19 patients with the clinical characteristics of this pathology when the patients had dental prostheses. Oral candidiasis is usually diagnosed using physical examination, clinical history, and exfoliative cytology with SBP or Pap staining [34,35,36]. However, the guide of the European Society of Clinical Microbiology and Infectious Diseases recommends cultivating the fungus in a specific medium for the diagnosis of *Candida* [37]. The recommendations made by the American Society for Infectious Diseases for the treatment of oropharyngeal candidiasis are to provide clotrimazole or nystatin as the first treatment, especially in patients who have not had the disease for a long period of time [38].

Like the Pap technique, molecular techniques were used to determine the presence of *Candida* subtypes in the patients studied. Consequently, seven species of *Candida* were identified in patients with diabetes and oral prostheses, unlike in Pap staining, which could only identify three patients with *Candida*. Nevertheless, the Pap technique is still used preferentially in dental consultations for the diagnosis of *Candida* in the Mexican population. A similar study was previously conducted in Monterrey, Mexico, in which *Candida* species were identified using culture media after collecting samples from the microbiology departments of each hospital participating in the study; *C. albicans*, *C. parapsilosis*, *C. tropicalis*, *C. glabrata*, *C. krusei*, and *C. guilliermondii* were mainly identified in a population studied from 2004 to 2007 [39].

Recent studies indicate an alarming increase in global *Candida* infections and drug resistance. For example, there are extensive reports of intrinsic and developed resistance to azole antifungals among several *Candida* species, including *C. albicans*, *C. parapsilosis, C. tropicalis*, *C. krusei*, and *C. glabrata*. Azole antifungals, such as fluconazole, are often the preferred treatment for many *Candida* infections [40,41]. Therefore, there is a need to seek new strategies for *Candida* diagnosis and treatment. Immunity or resistance involves the participation of the innate immune response through the TLR, DC-SIGN, galectin, and dectin receptors [42,43]. In addition, the pharmacological resistance of *Candida* has been shown to initially depend on the concentration and type of drug used as well as the immunological capacity of the infected individual [44]. Moreover, recent studies indicate that *Candida* has mutagenic capabilities that allow it to develop pharmacological resistance [45]. The development of resistance to treatments for candidiasis is mainly caused by the low pharmacological efficiency of such treatments to combat all *Candida* species observed during infection [46,47]. Given such resistance, the standard clinical approach to treating patients with candidiasis has been questioned, particularly given that the conventional diagnostic method, that is, by means of stains, does not confirm the *Candida* species, and therefore the treatment offered is not species-specific and may be ineffective. In contrast, molecular diagnosis allows for rapid identification of *Candida* species and the implementation of an appropriate treatment that can help avoid the systemic damage that is caused by the advanced contagion of species that typically develop resistance.

The damage caused by *Candida* in the oral epithelium provokes an inflammatory response that causes the release of inflammatory mediators and the production of recognition patterns of molecules such as TLRs. Jianwei et al. (2018) [48] demonstrated the activation of TLR2 in the presence of *C. albicans*. Consistent with these findings, Shaoru (2004) [49] found that both *TLR2* and *TLR4* mRNA expressions were low in patients diagnosed with candidiasis who did not receive pharmacological treatment. Similarly, no significant differences in TLR4 expression were found between healthy subjects and those infected with *Candida.* In addition, Pei et al. (2019) found that subjects infected with *Candida* showed a downregulation of IL-1β [50]. Similar to these previous results, we found a significant decrease in the gene expression of *TLR2* in the candidiasis group. TLR4 showed a downward trend, although it did not present significance between the control group and the group with candidiasis. It would be interesting to increase the number of patients to see if the *TLRs* show a significant decrease. Because diabetes mellitus can be associated with the presence of more predominant types of *Candida*, the analysis of additional molecules involved in the immune response to each type of *Candida* species will facilitate the development of more effective pharmacological treatments.

Currently, the strategy applied for the clinical care of patients infected by *Candida* in Mexico lacks the specificity to identify all species that may be present in patients with candidiasis. Additionally, the general treatment provided to these patients leads to the development of pharmacological resistance by some *Candida* species. This favors the development of resistant species, which contributes to increasing the clinical complications that can compromise the lives of patients.

In this study, we identified several Candida genotypes in subjects with type 2 diabetes mellitus who attended dental consultations. It is interesting to observe that the *Candida guilliermondii* genotype predominates and that there is co-expression of genotypes. The TLR gene expression, as we have mentioned above, shows similarity with published works. However, in this study, the number of study subjects may be a limitation since it would be very interesting to know the behavior of other immune response molecules and observe if the behavior of the immune response molecules is different according to the candida genotypes present. Also, we observed how the pharmacological treatment was affected.

## 5. Conclusions

In conclusion, it is advisable to use molecular diagnostic tools to accurately determine the species of *Candida* present in each patient, which will allow the administration of treatment that guarantees the elimination of all *Candida* species present. Such changes will contribute to advancing individualized medicine in Mexico.

## Figures and Tables

**Table 1:** Clinical characteristics of adult subjects diagnosed with type 2 diabetes mellitus and candidiasis treated in the dental clinic of the CICS-UST.

Variables	Number of Patients
***Gender***	
Female	12 (66.6%)
Male	6 (33.3%)
Age (years), mean (SD)	63.3 ± 14.2
Smoking	2 (11.1%)
Non-smoking	16 (88.8%)
Hypertension	11 (61.1%)
No hypertension	7 (38.8%)
Type 1 stomatitis	11 (61.1%)
Type 2 stomatitis	7 (38.8%)
***Type of candidiasis (observation of clinical pattern)***	
Pseudomembranous	15 (83.3%)
Erythematous	3 (16.6%)
Glycated hemoglobin (HbA1c) mean (SD)	9.3 ± 2.7

**Table 2:** *Candida* species identified in the study subjects by endpoint PCR. Specific primers for each Candida genotype were used, as described in the Materials and Methods section. (+) presence of *Candida,* (−) absence of *Candida*, (PAP) Papanicolaou (Pap) staining.

*Candida*									
Patient	PAP	*Albicans*	*Glabrata*	*Parapsilosis*	*Tropicallis*	*Dublinensis*	*Krusei*	*Guilermondi*	*Lusitanie*
01	−	−	−	−	−	−	−	+	−
02	−	−	−	−	−	−	−	+	−
03	−	+	+	−	+	−	−	+	−
04	+	−	−	−	−	−	−	−	−
05	−	−	−	−	+	−	+	+	+
06	−	−	−	−	+	−	−	+	−
07	−	−	−	−	+	−	+	+	+
08	−	−	−	−	−	−	−	−	−
09	−	−	−	−	−	−	−	−	−
10	−	−	−	−	−	−	−	−	−
11	+	−	−	−	−	−	+	+	−
12	−	−	−	−	−	−	+	−	−
13	+	+	−	+	−	−	+	−	−
14	−	−	−	−	−	−	+	−	−
15	−	−	−	−	−	−	−	−	+
16	−	−	−	−	−	−	−	−	+
17	−	−	−	−	−	−	−	−	−
18	−	−	−	−	−	−	−	−	+

## Data Availability

The data presented in this study are available on request from the corresponding author.

## References

[1] Arendorf T.M., Walker D.M. (1980). The prevalence and intra-oral distribution of *Candida albicans* in man. Arch. Oral Biol..

[2] Martins N., Ferreira I.C.F.R., Barros L., Silva S., Henriques M. (2014). Candidiasis: Predisposing factors, prevention, diagnosis and alternative treatment. Mycopathologia.

[3] Pappas P.G., Kauffman C.A., Andes D.R., Clancy C.J., Marr K.A., Ostrosky-Zeichner L., Reboli A.C., Schuster M.G., Vazquez J.A., Walsh T.J. (2016). Clinical practice guideline for the management of candidiasis: 2016 update by the Infectious Diseases Society of America. Clin. Infect. Dis..

[4] Madhavan P., Jamal F., Chong P.P. (2011). Laboratory isolation and identification of *Candida* species. J. Appl. Sci..

[5] de Resende M.A., de Sousa L.V.N.F., de Oliveira R.C.B.W., Koga-Ito C.Y., Lyon J.P. (2006). Prevalence and antifungal susceptibility of yeasts obtained from the oral cavity of elderly individuals. Mycopathologia.

[6] Guimarães T., Nucci M., Mendonça J.S., Martinez R., Brito L.R., Silva N., Moretti M.L., Salomão R., Colombo A.L. (2012). Epidemiology and predictors of a poor outcome in elderly patients with candidemia. Int. J. Infect. Dis..

[7] Khosravi A.R., Yarahmadi S., Baiat M., Shokri H., Pourkabireh M. (2008). Factors affecting the prevalence of yeasts in the oral cavity of patients with diabetes mellitus. J. Mycol. Médicale.

[8] Tang H., Liu W., Lin H., Lai C. (2015). Epidemiology and prognostic factors of candidemia in elderly patients. Geriatr. Gerontol. Int..

[9] Calvet H.M., Yoshikawa T.T. (2001). Infections in diabetes. Infect. Dis. Clin. N. Am..

[10] Bhuyan L., Hassan S., Dash K.C., Panda A., Behura S.S., Ramachandra S. (2018). *Candida* species diversity in oral cavity of type 2 diabetic patients and their in vitro antifungal susceptibility. Contemp. Clin. Dent..

[11] King H., Aubert R.E., Herman W.H. (1998). Global burden of diabetes, 1995–2025: Prevalence, numerical estimates, and projections. Diabetes Care.

[12] Agarwal S., Raman R., Paul P.G., Rani P.K., Uthra S., Gayathree R., McCarty C., Kumaramanickavel G., Sharma T. (2005). Sankara Nethralaya—Diabetic retinopathy epidemiology and molecular genetic study (SN—DREAMS 1): Study design and research methodology. Ophthalmic Epidemiol..

[13] Davenport J.C. (1970). The oral distribution of *Candida* in denture stomatitis. Br. Dent. J..

[14] Kadir T., Pisiriciler R., Akyüz S., Yarat A., Emekli N., Ipbüker A. (2002). Mycological and cytological examination of oral candidal carriage in diabetic patients and non-diabetic control subjects: Thorough analysis of local aetiologic and systemic factors. J. Oral Rehabil..

[15] Duggan S., Essig F., Hünniger K., Mokhtari Z., Bauer L., Lehnert T., Brandes S., Häder A., Jacobsen I.D., Martin R. (2015). Neutrophil activation by *Candida glabrata* but not *Candida albicans* promotes fungal uptake by monocytes. Cell Microbiol..

[16] Wilson R.M., Reeves W.G. (1986). Neutrophil phagocytosis and killing in insulin-dependent diabetes. Clin. Exp. Immunol..

[17] Pinto E., Ribeiro I.C., Ferreira N.J., Fortes C.E., Fonseca P.A., Figueiral M.H. (2008). Correlation between enzyme production, germ tube formation and susceptibility to fluconazole in *Candida* species isolated from patients with denture-related stomatitis and control individuals. J. Oral. Pathol. Med..

[18] Motta-Silva A.C., Aleva N.A., Chavasco J.K., Armond M.C., França J.P., Pereira L.J. (2010). Erythematous oral candidiasis in patients with controlled type II diabetes mellitus and complete dentures. Mycopathologia.

[19] Calderone R.A., Fonzi W.A. (2001). Virulence factors of *Candida albicans*. Trends Microbiol..

[20] Balan P., Gogineni S.B., Kumari S., Shetty V., Rangare A.L., Castelino R.L., Areekat K F. (2015). *Candida* carriage rate and growth characteristics of saliva in diabetes mellitus patients: A case-control study. J. Dent. Res. Dent. Clin. Dent. Prospect..

[21] Nowakowska D., Kurnatowska A., Stray-Pedersen B., Wilczyński J. (2004). Species distribution and influence of glycemic control on fungal infections in pregnant women with diabetes. J. Infect..

[22] Dorko E., Baranova Z., Jenča A., Kizek P., Pilipčinec E., Tkáčiková L. (2005). Diabetes mellitus and candidiases. Folia Microbiol..

[23] Feller L., Khammissa R.A.G., Chandran R., Altini M., Lemmer J. (2014). Oral candidosis in relation to oral immunity. J. Oral Pathol. Med..

[24] Rós Ásmundsdóttir L., Erlendsdóttir H., Haraldsson G., Guo H., Xu J., Gottfredsson M. (2008). Molecular epidemiology of candidemia: Evidence of clusters of smoldering nosocomial infections. Clin. Infect. Dis..

[25] Moosazadeh M., Akbari M., Tabrizi R., Ghorbani A., Golkari A., Banakar M., Sekhavati E., Kavari S.H., Lankarani K.B. (2016). Denture stomatitis and *Candida albicans* in Iranian population: A systematic review and meta-analysis. J. Dent..

[26] Netea M.G., Joosten L.A.B., Van Der Meer J.W.M., Kullberg B.J., Van De Veerdonk F.L. (2015). Immune defence against *Candida* fungal infections. Nat. Rev. Immunol..

[27] Netea M.G., Brown G.D., Kullberg B.J., Gow N.A.R. (2008). An integrated model of the recognition of *Candida albicans* by the innate immune system. Nat. Rev. Microbiol..

[28] Brown G.D., Gordon S. (2001). A new receptor for β-glucans. Nature.

[29] Cambi A., Gijzen K., de Vries I.J.M., Torensma R., Joosten B., Adema G.J., Netea G., Kullberg B.-J., Romani L., Figdor G. (2003). The C-type lectin DC-SIGN (CD209) is an antigen-uptake receptor for *Candida albicans* on dendritic cells. Eur. J. Immunol..

[30] Netea M.G., Gow N.A.R., Munro C.A., Bates S., Collins C., Ferwerda G., Hobson R.P., Bertram G., Hughes H.B., Jansen T. (2006). Immune sensing of *Candida albicans* requires cooperative recognition of mannans and glucans by lectin and Toll-like receptors. J. Clin. Investig..

[31] Jaeger M., Carvalho A., Cunha C., Plantinga T.S., Van De Veerdonk F., Puccetti M., Galosi C., Joosten L.A.B., Dupont B., Kullberg B.J. (2016). Association of a variable number tandem repeat in the NLRP3 gene in women with susceptibility to RVVC. Eur. J. Clin. Microbiol. Infect. Dis..

[32] Jaeger M., van der Lee R., Cheng S.-C., Johnson M.D., Kumar V., Ng A., Plantinga T.S., Smeekens S.P., Oosting M., Wang X. (2015). The RIG-I-like helicase receptor MDA5 (IFIH1) is involved in the host defense against *Candida* infections. Eur. J. Clin. Microbiol. Infect. Dis..

[33] Groslambert M., Py B.F. (2018). Spotlight on the NLRP3 inflammasome pathway. J. Inflamm. Res..

[34] Picciani B.L.S., Michalski-Santos B., Carneiro S., Sampaio A.L., Avelleira J.C.R., Azulay D.R., Pinto J.M.N., Dias E.P. (2013). Oral candidiasis in patients with psoriasis: Correlation of oral examination and cytopathological evaluation with psoriasis disease severity and treatment. J. Am. Acad. Dermatol..

[35] Miller D.J. (2002). Diagnosis and management of *Candida* and other fungal infections of the head and neck. Curr. Infect. Dis. Rep..

[36] Hellstein J.W., Marek C.L. (2019). Candidiasis: Red and white manifestations in the oral cavity. Head Neck Pathol..

[37] Cuenca-Estrella M., Verweij P.E., Arendrup M.C., Arikan-Akdagli S., Bille J., Donnelly J.P., Jensen H.E., Lass-Flörl C., Richardson M.D., Akova M. (2012). ESCMID* guideline for the diagnosis and management of *Candida* diseases 2012: Diagnostic procedures. Clin. Microbiol. Infect..

[38] Pappas P.G., Kauffman C.A., Andes D., Benjamin D.K., Calandra T.F., Edwards J.E., Filler S.G., Fisher J.F., Kullberg B.J., Ostrosky-Zeichner L. (2009). Clinical practice guidelines for the management of candidiasis: 2009 update by the Infectious Diseases Society of America. Clin. Infect. Dis..

[39] González G.M., Elizondo M., Ayala J. (2008). Trends in species distribution and susceptibility of bloodstream isolates of *Candida* collected in Monterrey, Mexico, to seven antifungal agents: Results of a 3-year (2004 to 2007) surveillance study. J. Clin. Microbiol..

[40] Srivastava V., Singla R.K., Dubey A.K. (2018). Emerging virulence, drug resistance and future anti-fungal drugs for *Candida* pathogens. Curr. Top. Med. Chem..

[41] Whaley S.G., Berkow E.L., Rybak J.M., Nishimoto A.T., Barker K.S., Rogers P.D. (2017). Azole antifungal resistance in *Candida albicans* and emerging non-albicans *Candida* species. Front. Microbiol..

[42] Cheng S.C., Joosten L.A.B., Kullberg B.J., Netea M.G. (2012). Interplay between *Candida albicans* and the mammalian innate host defense. Infect. Immun..

[43] Richardson J.P., Moyes D.L. (2015). Adaptive immune responses to *Candida albicans* infection. Virulence.

[44] Wuyts J., Van Dijck P., Holtappels M. (2018). Fungal persister cells: The basis for recalcitrant infections?. PLoS Pathog..

[45] Lockhart S.R., Ghannoum M.A., Alexander B.D. (2017). Establishment and use of epidemiological cutoff values for molds and yeasts by use of the clinical and laboratory standards institute M57 standard. J. Clin. Microbiol..

[46] Pristov K.E., Ghannoum M.A. (2019). Resistance of *Candida* to azoles and echinocandins worldwide. Clin. Microbiol. Infect..

[47] Berman J., Krysan D.J. (2020). Drug resistance and tolerance in fungi. Nat. Rev. Microbiol..

[48] Liu J., Geng F., Sun H., Wang X., Zhang H., Yang Q., Zhang J. (2018). *Candida albicans* induces TLR2/MyD88/NF-κB signaling and inflammation in oral lichen planus-derived keratinocytes. J. Infect. Dev. Ctries..

[49] Shaoru Z., Jiawen L., Xuesong J., Yanqing W. (2004). The expression of toll-like receptor 2 and 4 mRNA in local tissues of model of oropharyngeal candidiasis in mice. J. Huazhong Univ. Sci. Technol. [Med. Sci.].

[50] Ye P., Wang X., Ge S., Chen W., Wang W., Han X. (2019). Long-term cigarette smoking suppresses NLRP3 inflammasome activation in oral mucosal epithelium and attenuates host defense against *Candida albicans* in a rat model. Biomed. Pharmacother..

